# Imaging Study of MnO_2_-Based Nanomotors Modulating HIF-1α/Lipid Droplet Biogenesis and Activating the cGAS-STING Pathway

**DOI:** 10.3390/bios16050261

**Published:** 2026-05-01

**Authors:** Ziyi Li, Yingxin Tian, Gefei Ren, Yingshu Guo

**Affiliations:** School of Chemistry and Chemical Engineering, Qilu University of Technology (Shandong Academy of Sciences), Jinan 250353, China

**Keywords:** nanomotor, hypoxia alleviation, lipid droplets, cGAS-STING pathway, imaging analysis

## Abstract

The overexpression of hypoxia-inducible factor-1α (HIF-1α) suppresses STING signaling and modulates lipid metabolism in tumor cells, leading to abnormal lipid droplet (LD) accumulation. Herein, we constructed a manganese dioxide (MnO_2_)-based nanomotor (HMIP@A). HMIP@A depletes intracellular hydrogen peroxide (H_2_O_2_) and glutathione (GSH) to generate oxygen (O_2_), reactive oxygen species (ROS), and manganese (Mn^2+^). A dual strategy of “oxygen supplementation” and “small-molecule inhibition” synergistically downregulates HIF-1α, thereby suppressing LD biogenesis. This process sensitizes tumor cells to ROS, leading to severe DNA damage. Released Mn^2+^ and damaged DNA synergistically activate the cyclic GMP-AMP synthase (cGAS)-stimulator of interferon genes (STING) pathway. In vitro, HMIP@A markedly increases ROS production, lipid peroxidation (LPO), and DNA damage, thereby inducing tumor cell death, immunogenic cell death (ICD), and dendritic cell (DC) maturation. Furthermore, HMIP@A exhibits excellent penetration in tumor spheroids. Overall, this study provides a theoretical basis for the design of nanomedicines through a strategy integrating metabolic intervention, oxidative damage sensitization, and immune activation.

## 1. Introduction

Immunogenic cell death (ICD), as a specific form of regulated cell death that enables the release of tumor-associated antigens and the activation of dendritic cells, serves as a crucial bridge linking local therapy with systemic immunity. However, the ubiquitous hypoxic tumor microenvironment severely impedes this process. Hypoxia-inducible factor-1α (HIF-1α) is the key transcription factor that drives the reprogramming of lipid metabolism in tumor cells, directly leading to the abnormal accumulation of lipid droplets (LDs) [1]. LDs are organelles enclosed by a phospholipid monolayer that store neutral lipids. They not only supply energy but also sequester oxidizable polyunsaturated fatty acids and scavenge reactive oxygen species (ROS), thereby constituting a critical “intracellular antioxidant buffer pool” [2]. This property renders LDs a vital protective mechanism for tumor cells to resist therapies such as chemotherapy, radiotherapy, and chemodynamic therapy (CDT) [3]. Therefore, the targeted depletion of LDs can not only deprive tumor cells of their energy supply but, more importantly, disrupt their antioxidant defense systems, rendering tumor cells more susceptible to oxidative damage, which represents a promising therapeutic strategy.

Nanomaterials have been widely used due to their unique size effects, surface effects, and enzyme-like activities [4]. Manganese dioxide (MnO_2_) nanomaterials can decompose endogenous hydrogen peroxide (H_2_O_2_) within tumor cells into oxygen (O_2_), thereby alleviating tumor hypoxia [5,6]. Concurrently, MnO_2_ nanomaterials are reduced to Mn^2+^ by glutathione (GSH) [7,8]. The generated Mn^2+^ not only catalyzes the production of hydroxyl radicals (·OH) via Fenton-like reactions [9,10,11,12] but also activates the cyclic GMP-AMP synthase (cGAS)-stimulator of interferon genes (STING) pathway, thereby inducing ICD [13]. These findings have increasingly promoted the use of MnO_2_-based nanomaterials as functional carriers against tumor cells [14,15].

Nanomotors achieve self-propulsion by converting diverse energy sources into kinetic energy [16]. These systems are generally driven by asymmetric chemical reactions on their surfaces [17,18] or actuated by external fields [19,20]. For chemically powered nanomotors, H_2_O_2_ serves as a primary endogenous fuel, which can be catalytically decomposed to generate propulsive bubbles [21]. Notably, owing to their intrinsic catalytic activity toward H_2_O_2_, MnO_2_ nanomaterials have emerged as promising platforms for the construction of self-propelled nanomotor systems [22,23].

Aptamers are short, single-stranded DNA or RNA molecules that fold into unique three-dimensional structures through intramolecular interactions, enabling high-affinity and high-specificity binding to target molecules. Owing to their excellent thermal stability, low immunogenicity, efficient tissue uptake, high sensitivity, and cost-effectiveness, aptamers are widely used for tumor targeting [24]. Notably, certain aptamers bind to nucleolin receptors overexpressed on tumor cell membranes [25], thereby enhancing cellular uptake.

**Scheme 1:**
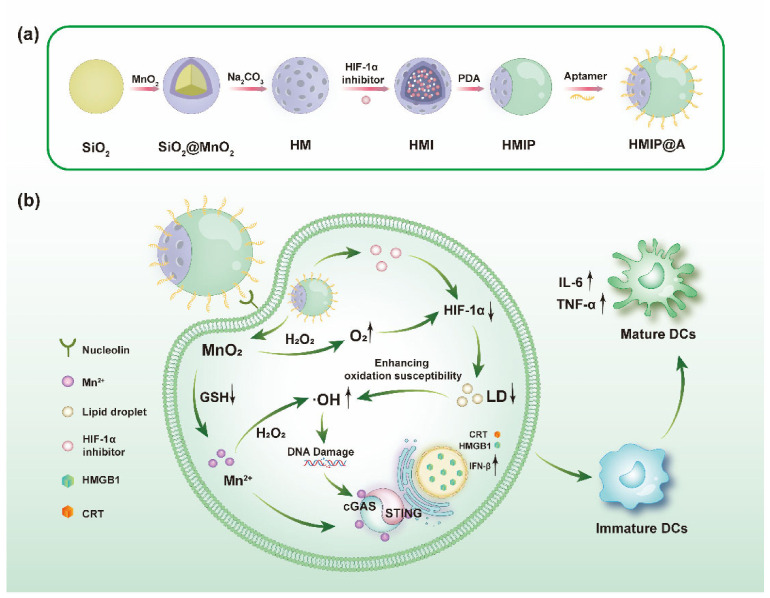
(**a**) Schematic illustration of the preparation of HMIP@A. (**b**) Schematic illustration of the mechanism of HMIP@A in modulating HIF-1α/lipid droplet biogenesis and activating the cGAS-STING pathway. Upward and downward arrows represent increase and decrease, respectively.

Motivated by these findings, we report an intelligent nanomotor that enables a cascade from metabolic intervention to immune activation at the cellular level. This nanomotor is based on MnO_2_ nanomaterials as the core carrier and incorporates a specific small-molecule HIF-1α inhibitor. To endow active motion, an asymmetric polydopamine (PDA) coating was constructed on its surface [20,26,27]. Subsequently, aptamers were conjugated to obtain the final HMIP@A nanomotors (Scheme 1a). This design integrates multiple synergistic mechanisms. First, the aptamer targets nucleolin receptors overexpressed on 4T1 cell membranes, thereby enhancing cellular uptake. Second, the MnO_2_ exhibits catalase-like activity, decomposing endogenous H_2_O_2_ to generate O_2_. The generated O_2_ not only drives self-propulsion but also alleviates hypoxia in tumor cells, thereby contributing to HIF-1α downregulation. However, due to rapid oxygen consumption in tumor cells, hypoxia alleviation via catalytic O_2_ generation alone remains insufficient. Therefore, a targeted HIF-1α inhibitor is incorporated. A dual strategy combining “oxygen supplementation” and “small-molecule inhibition” synergistically suppresses HIF-1α, thereby inhibiting lipid droplet biogenesis and enhancing tumor cell sensitivity to reactive oxygen species ROS [2,28]. Furthermore, MnO_2_ reacts with intracellular GSH to produce Mn^2+^ [29], which catalyzes H_2_O_2_ to generate ·OH, leading to severe DNA damage. Concurrently, the cGAS-STING pathway is activated, which, together with the oxidative damage, induces ICD (Scheme 1b).

## 2. Materials and Methods

### 2.1. Materials

Ammonium hydroxide (NH_3_·H_2_O, 28%) and hydrogen peroxide (H_2_O_2_, 30 wt% solution) were purchased from Sinopharm Chemical Reagent Co., Ltd. (Shanghai, China). Potassium permanganate (KMnO_4_) was obtained from Yantai Yuandong Fine Chemicals Co., Ltd. (Yantai, China). Tetraethyl orthosilicate (TEOS), isopropanol, anhydrous ethanol, dopamine hydrochloride, sodium carbonate anhydrous (Na_2_CO_3_), and Rhodamine B were purchased from Shanghai Macklin Biochemical Technology Co., Ltd. (Shanghai, China). The HIF-1α inhibitor, (3-aminopropyl)triethoxysilane (APTES), 1-ethyl-3-(3-dimethylaminopropyl)carbodiimide hydrochloride (EDC), N-hydroxysuccinimide (NHS), polyacrylic acid, and cobalt(II) chloride (CoCl_2_) were purchased from Shanghai Aladdin Biochemical Technology Co., Ltd. (Shanghai, China). Mouse mammary carcinoma cells (4T1) were obtained from Wuhan Zishan Biotechnology Co., Ltd. (Wuhan, China). Mouse bone marrow-derived dendritic cells (DC2.4), breast cancer cells (MDA-MB-231), and mouse embryonic fibroblasts (NIH/3T3) were purchased from Wuhan Boster Biological Technology Co., Ltd. (Wuhan, China). Penicillin–streptomycin solution (100×) and fetal bovine serum (FBS) were obtained from Wuhan Pricella Life Science & Technology Co., Ltd. (Wuhan, China). PMI 1640 medium was purchased from Wuhan Servicebio Technology Co., Ltd. (Wuhan, China). Tris-buffered saline with Tween 20 (TBST), phosphate-buffered saline (PBS), 2,2-biquinoline-4,4-dicarboxylic acid disodium salt (BCA) protein assay kit, enhanced chemiluminescence (ECL) reagent, and trypsin digestion solution were purchased from Suzhou NCM Biotech Co., Ltd. (Suzhou, China). A multicolor prestained protein molecular weight marker was obtained from Shanghai Epizyme Biotech Co., Ltd. (Shanghai, China). Methylene blue (MB, ≥90%) and 5,5′-dithiobis-(2-nitrobenzoic acid) (DTNB, ≥99%) were obtained as indicated. C11-BODIPY 581/591 assay kit, Annexin V-FITC/PI apoptosis detection kit, 4′,6-diamidino-2-phenylindole (DAPI) staining solution, Calcein-AM/PI cell activity kit, MTT cytotoxicity assay kit, and anti-CRT antibody were purchased from Shanghai Beyotime Biotechnology Co., Ltd. (Shanghai, China). Ferrostatin-1 (Fer-1) was obtained from Ambeed, Inc. (Buffalo Grove, IL, USA). Nile Red and RU.521 were obtained from MedChemExpress LLC (Monmouth Junction, NJ, USA). Horseradish peroxidase (HRP)-conjugated goat anti-rabbit IgG, HRP-conjugated goat anti-mouse IgG, and anti-HIF-1α antibody were purchased from Shanghai Abways Biotechnology Co., Ltd. (Shanghai, China). The APC-CD86 antibody and FITC-CD80 antibody were obtained from Suzhou 4A Biotech Co., Ltd. (Suzhou, China). The anti-cGAS antibody, anti-p-IRF3 antibody, anti-HMGB1 antibody, iFluor™ 488-conjugated IgG antibody, TNF-α ELISA kit, and IL-6 ELISA kit were purchased from Hangzhou HuaAn Biotechnology Co., Ltd. (Hangzhou, China). The anti-p-TBK1 antibody was purchased from Immunoway Biotechnology Co., Ltd. (San Jose, CA, USA). The reactive oxygen species assay kit, GSH assay kit, and β-actin antibody were obtained from Beijing Solarbio Science & Technology Co., Ltd. (Beijing, China). The carboxyl-modified aptamer (5′-COOH-GGTGGTGGTGGTTGTGGTGGTGGTGG-3′) was synthesized by Sangon Biotech (Shanghai) Co., Ltd. (Shanghai, China).

### 2.2. Instruments

Size and zeta potential data were collected using a laser particle size analyzer (Zetasizer Nano ZS90, Malvern Instrument Ltd., Malvern, UK). Ultraviolet-visible absorption spectra were obtained using a UV-visible spectrophotometer (Cary 60, Agilent Technologies, Inc., Penang, Malaysia). Fluorescence images were obtained using a fluorescence microscope (Nikon Ti2-E, Nikon Corporation, Tokyo, Japan). Transmission electron microscopy (TEM) images were recorded using a transmission electron microscope (JEOL JEM-F200, Japan Electron Optics Ltd., Tokyo, Japan). The manganese (Mn) content was determined using an inductively coupled plasma optical emission spectrometer (ICP-OES; Avio 200, PerkinElmer, Shelton, CT, USA). Fluorescence images of tumor spheroids were acquired using a confocal laser scanning microscope (CLSM; Leica SP8, Leica Microsystems, Wetzlar, Germany). Fluorescence intensity was measured using flow cytometry (CytoFLEX, Beckman Coulter, Suzhou, China). MTT assay results were measured using a microplate reader (K3 Touch, Thermo Scientific, Shanghai, China). The protein bands were visualized with a fluorescence and chemiluminescence imaging system. (ChemiScope 6200, Clinx Science Instruments Co., Ltd., Shanghai, China).

### 2.3. Preparation of HMIP@A

Synthesis of HM: First, 140 mL of absolute ethanol, 20 mL of deionized water, and 5 mL of aqueous ammonia (28%) were added to a 250 mL round-bottom flask. The mixture was stirred at 1000 rpm and 45 °C for 30 min. Subsequently, 5 mL of TEOS was added dropwise, and the reaction was stirred at 45 °C for 3 h. The resulting products were washed three times with absolute ethanol via centrifugation (12,000 rpm, 15 min). After vacuum drying at 45 °C for 4 h, silica nanoparticles (SiO_2_) were obtained. Next, 300 mg of potassium permanganate was dissolved in 10 mL of deionized water. Then, 10 mL of a SiO_2_ dispersion (4 mg/mL) was added dropwise under ultrasonication. After sonication for 6 h, the mixture was centrifuged at 12,000 rpm for 10 min. The collected precipitate was washed with deionized water by centrifugation (12,000 rpm, 10 min) until the supernatant became colorless, yielding MnO_2_-coated silica nanoparticles (SiO_2_@MnO_2_). Finally, 0.1 g of SiO_2_@MnO_2_ was dispersed in 50 mL of deionized water, followed by the addition of anhydrous sodium carbonate (10.6 g). The mixture was stirred at 60 °C for 12 h and then centrifuged at 12,000 rpm for 10 min. The precipitate was washed three times with deionized water (12,000 rpm, 10 min) to obtain hollow manganese dioxide nanoparticles (HM).

Synthesis of HMI: The as-prepared HM (1 mL, 1 mg/mL) was mixed with a HIF-1α inhibitor solution (1 mL, 1 mg/mL) and stirred at 4 °C for 4 h. The mixture was then centrifuged at 12,000 rpm for 10 min. The precipitate was washed three times with deionized water (12,000 rpm, 10 min) to obtain HIF-1α inhibitor-loaded HM nanoparticles (HMI).

Synthesis of HMIP: First, 20 mg of HMI was dispersed in 15 mL of absolute ethanol, followed by the addition of 50 μL of APTES. After stirring at room temperature for 6 h, the mixture was centrifuged at 12,000 rpm for 10 min. The precipitate was washed three times with deionized water and absolute ethanol alternately to obtain aminated HMI (HMI-NH_2_). Subsequently, 5 mg of HMI-NH_2_ was dispersed in 7 mL of deionized water. Aqueous ammonia (28%, 50 μL) and polyacrylic acid (500 μg/mL, 100 μL) were sequentially added. The mixture was sonicated for 30 min and then stirred at room temperature for 20 min. Then, 30 mL of isopropanol was added dropwise, followed by the addition of 2 mL of dopamine hydrochloride solution (500 μg/mL). The reaction was stirred at 50 °C in the dark for 3.5 h and then centrifuged at 12,000 rpm for 10 min. The precipitate was washed three times with deionized water and absolute ethanol alternately and vacuum-dried at 50 °C to obtain the PDA asymmetrically coated HMI nanomotors (HMIP).

Synthesis of HMIP@A: The carboxyl-modified aptamer was activated via a carbodiimide coupling reaction. Briefly, 400 μL of the carboxyl-modified aptamer (5 μM) was mixed with 50 μL of EDC (0.2 mM) and 125 μL of NHS (0.2 mM) and stirred for 30 min to activate the carboxyl groups. Subsequently, 500 μL of HMIP (1 mg/mL) was added, and the mixture was stirred at 500 rpm at room temperature for 4 h [30,31]. The products were washed three times with PBS by centrifugation (12,000 rpm, 10 min) and then lyophilized to obtain aptamer-conjugated nanomotors (HMIP@A).

Synthesis of HMI@A: The synthesis procedure for HMI@A was the same as that for HMIP@A, except that HMIP was replaced with an equivalent mass of HMI.

### 2.4. Cell Culture

4T1 and DC2.4 cells were cultured in RPMI 1640 medium supplemented with 10% FBS and 1% penicillin–streptomycin solution (100×). To obtain H-4T1 cells, 4T1 cells were pretreated with a CoCl_2_ solution (80 μM) for 4 h [32,33,34]. NIH/3T3 and MDA-MB-231 cells were cultured in DMEM supplemented with 10% FBS and 1% penicillin–streptomycin solution (100×). All cells were maintained at 37 °C in a humidified atmosphere containing 5% CO_2_.

### 2.5. Enhanced Penetration of 4T1 Tumor Spheroids

NIH/3T3 and 4T1 cells (1200 cells total; 4:1 ratio) were seeded into U-bottom plates and cultured for 5 days to form tumor spheroids. For Rhodamine B (RhB) labeling, the HMI@A and HMIP@A nanoparticles were incubated with RhB solution for 12 h to obtain RhB-labeled HMI@A (RhB/HMI@A) and HMIP@A (RhB/HMIP@A). The spheroids were then incubated with these nanoparticles for 12 h. Images were captured using confocal laser scanning microscopy (CLSM) (Leica SP8, Leica Microsystems, Wetzlar, Germany). Image analysis was performed using the Leica Application Suite X Software.

### 2.6. Monitoring Intracellular ROS, GSH, Lipid Peroxidation, and Lipid Droplets

Intracellular ROS Assessment: H-4T1 cells were used to evaluate intracellular ROS levels. Cells were treated with PBS, HM, HMI, HMIP, or HMIP@A (vs. C_HM_ = 50 μg/mL) for 6 h at 37 °C. After treatment, the cells were washed three times with PBS. Subsequently, the DCFH-DA working solution was added, and the cells were incubated in the dark for 20 min. After washing with PBS, intracellular ROS levels were visualized using fluorescence microscopy. (Nikon Ti2-E, Nikon Corporation, Tokyo, Japan). Image analysis was performed using the NIS-Elements AR 3.2 software.

Intracellular GSH Assay: H-4T1 cells were treated with PBS, HM, HMI, HMIP, or HMIP@A (vs. C_HM_ = 50 μg/mL) in 2 mL of medium for 6 h at 37 °C with 5% CO_2_. After treatment, the cells were harvested by trypsin digestion and centrifugation. The collected cells were lysed by repeated freeze–thaw cycles between liquid nitrogen and a 37 °C water bath, and the intracellular GSH content was determined using a GSH assay kit according to the manufacturer’s instructions.

Lipid Peroxidation Assay: H-4T1 cells were treated with PBS, HM, HMI, HMIP, or HMIP@A (vs. C_HM_ = 80 μg/mL) in 2 mL of medium for 6 h at 37 °C. After treatment, the cells were washed three times with PBS. The cells were then incubated with a lipid peroxidation assay kit according to the manufacturer’s instructions and washed three times with PBS. Intracellular lipid peroxidation levels were visualized using fluorescence microscopy.

Lipid Droplet Imaging: H-4T1 cells were treated with PBS, HM, HMI, HMIP, or HMIP@A (vs. C_HM_ = 80 μg/mL) in 2 mL of medium for 6 h at 37 °C. After treatment, the cells were washed three times with PBS. Next, 200 μL of Nile Red solution (10 μg/mL) was added, and the cells were incubated at 37 °C for 30 min. After washing three times with PBS, 1 mL of medium containing DAPI working solution was added, followed by incubation at 37 °C for 10 min. The cells were then washed three times with PBS, and intracellular lipid droplets were visualized using fluorescence microscopy.

### 2.7. Western Blot Analysis

Cells were treated with PBS, HM, HMI, HMIP, or HMIP@A (vs. C_HM_ = 100 μg/mL) in 2 mL of medium for 24 h at 37 °C. After treatment, the cells were washed three times with PBS, and 150 μL of SDS lysis buffer was added. The lysates were centrifuged at 12,000 rpm for 5 min, and the supernatant was collected. Protein concentration was determined using a BCA protein assay kit. Next, 100 μL of each protein sample was mixed with 20 μL of SDS-PAGE sample loading buffer (6×) and boiled for 5–10 min. Based on the quantitation results, 20–40 μg of protein per well was loaded onto a 10% or 12% SDS-PAGE gel, together with 4–6 μL of a multicolor prestained protein marker. Proteins were separated by vertical electrophoresis at 80 V for 1–2 h and then transferred onto polyvinylidene fluoride (PVDF) membranes via electroblotting at 120 mA for 1 h. The membranes were blocked with 5% non-fat milk in TBST buffer (25 mL) for 1 h at room temperature and washed three times with TBST (10 min each). Membranes were then incubated overnight at 4 °C with primary antibodies against HIF-1α, cGAS, p-IRF3, p-TBK1, and β-actin. After washing three times with TBST, the PVDF membranes were incubated with HRP-conjugated goat anti-rabbit IgG or anti-mouse IgG (1:2000) for 1 h at room temperature. After three additional washes with TBST, the protein bands were developed using a chemiluminescent substrate and visualized with a fluorescence and chemiluminescence imaging system. (ChemiScope 6200, Clinx Science Instruments Co., Ltd., Shanghai, China). Image analysis was performed using the ChemiScope Capture software.

### 2.8. Assessment of Dendritic Cell Maturation

At the cellular level, DC2.4 cell maturation induced by HMIP@A was evaluated. H-4T1 cells were treated with PBS, HM, HMI, HMIP, or HMIP@A (vs. C_HM_ = 100 μg/mL) in 2 mL of medium for 12 h. The cell culture supernatants were then collected and utilized to culture DC2.4 cells for an additional 12 h. The treated DC2.4 cells were harvested and co-incubated with APC-CD86 and FITC-CD80 antibodies at 4 °C for 20 min. After washing with PBS, the cells were analyzed via flow cytometry.

## 3. Results and Discussion

### 3.1. Characterizations of HMIP@A

**Figure 1:**
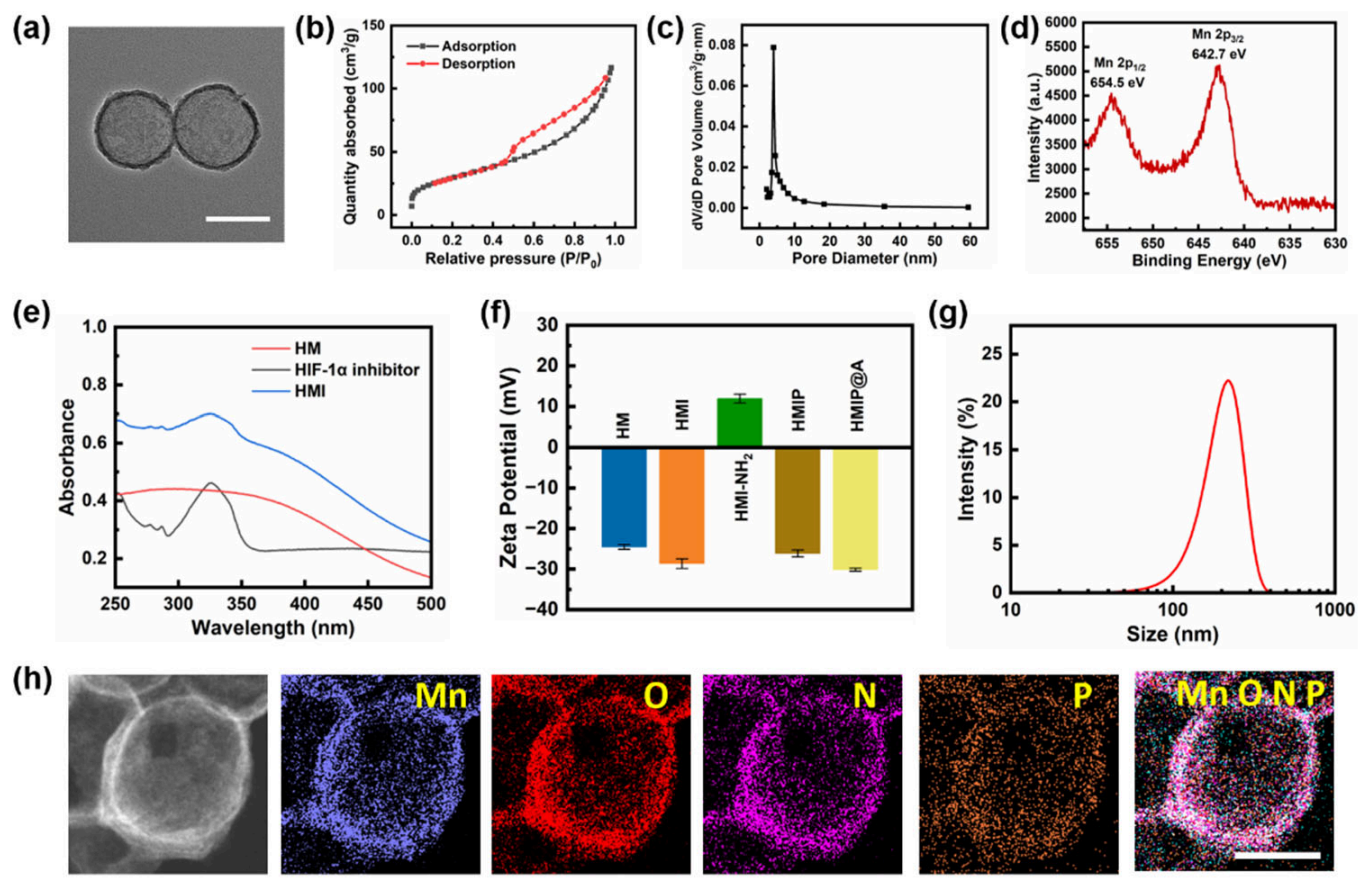
(**a**) TEM image of HM, scale bar = 100 nm. (**b**) N_2_ adsorption–desorption isotherm of HM. (**c**) Pore size distribution of HM. (**d**) Mn 2p XPS spectra of HM. (**e**) UV-vis absorption spectra of HM, HIF-1α inhibitor, and HMI. (**f**) Zeta potentials of HM, HMI, HMI-NH_2_, HMIP, and HMIP@A. (**g**) Hydrodynamic diameter of HMIP@A. (**h**) TEM-mapping images of HMIP@A, scale bar = 100 nm.

TEM imaging revealed that the HM nanoparticles possessed a distinct hollow structure (Figure 1a). The BET surface area was determined to be 107.53 m^2^/g (Figure 1b), with an average pore size of approximately 6 nm (Figure 1c), providing a structural basis for small-molecule loading [35]. XPS analysis showed two characteristic peaks at 654.5 eV and 642.7 eV corresponding to Mn 2p_1_/_2_ and Mn 2p_3_/_2_, respectively (Figure 1d) [36,37,38,39,40]. Furthermore, HMI exhibited an absorption peak at ~328 nm [1,41] (Figure 1e), consistent with the characteristic absorption of the HIF-1α inhibitor. The zeta potential of the aminated HMI exhibited a positive zeta potential (Figure 1f). The hydrodynamic diameter of HMIP@A was mainly distributed in the range of ~110–400 nm (Figure 1g). In addition, TEM elemental mapping images clearly revealed the asymmetric coating structure and the distribution of Mn, O, N, and P elements (Figure 1h). The presence of phosphorus (P) confirmed the successful conjugation of the aptamer onto HMIP. Collectively, these results verify the successful construction of HMIP@A. Finally, the hydrodynamic size of HMIP@A in PBS was monitored over time (Figure S1). The negligible changes in both the hydrodynamic size and the polydispersity index (PDI) indicate the excellent colloidal stability of the HMIP@A nanomotors.

**Figure 2:**
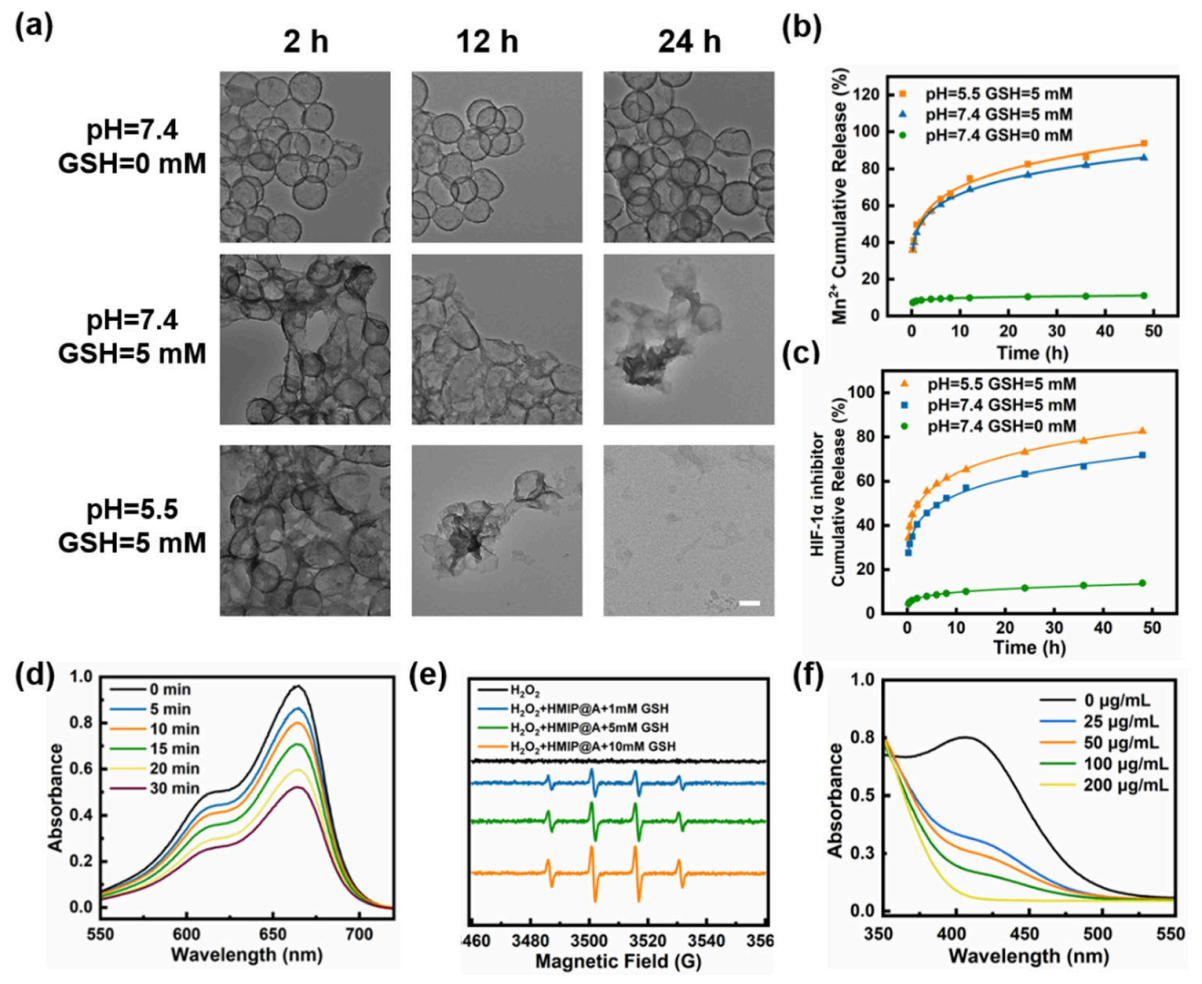
(**a**) TEM images of degraded HM, scale bar = 100 nm. Cumulative release curves of (**b**) Mn^2+^ and (**c**) HIF-1α inhibitor, *n* = 3. (**d**) UV-vis absorption spectra of MB incubated with HMIP@A. (**e**) ESR spectra of the reaction systems using DMPO as the spin trap. (**f**) UV-vis absorption spectra of DTNB incubated with different concentrations of HMIP@A by adding GSH.

The degradation behavior of HMIP@A in different pH and GSH environments was systematically investigated. TEM images revealed that incubation at pH 5.5 in the presence of 5 mM GSH led to significant collapse of the hollow structure of HM nanoparticles (Figure 2a), confirming their dual pH/GSH-responsive degradation. Under these conditions, the cumulative release of Mn^2+^ and HIF-1α inhibitor increased to 85.91% (Figure 2b) and 82.60% (Figure 2c), respectively, which were significantly higher than those observed under neutral conditions.

Leveraging the Mn^2+^-mediated Fenton-like reaction, the ability of HMIP@A to catalyze the generation of ·OH was evaluated. The generation of ·OH was first indirectly assessed using the methylene blue (MB) degradation assay [8]. As shown in Figure 2d, the intensity of the MB absorption peak at ~665 nm decreased continuously with reaction time, indicating oxidative degradation. This effect arises from Mn^2+^ released from HMIP@A upon reaction with GSH, which subsequently reacts with H_2_O_2_ to produce highly reactive ·OH, driving MB degradation. Furthermore, the formation of ·OH was directly confirmed using 5,5-dimethyl-1-pyrroline N-oxide (DMPO) as a spin-trapping agent. ESR spectroscopy revealed a characteristic 1:2:2:1 quartet signal corresponding to the DMPO ·OH adduct (Figure 2e) [42,43,44], confirming ·OH generation. Notably, the ·OH signal increased with GSH concentration, reflecting that GSH promotes Mn^2+^ release and thereby amplifies the Fenton-like reaction, leading to enhanced ·OH generation [45].

Furthermore, the ability of HMIP@A to deplete GSH was evaluated using 5,5′-dithiobis(2-nitrobenzoic acid) (DTNB) as an indicator [46]. GSH reduces DTNB to 2-nitro-5-thiobenzoic acid (TNB), which exhibits a characteristic absorption at ~412 nm. As shown in Figure 2f, the absorbance at ~412 nm decreased in a concentration-dependent manner with increasing HMIP@A concentration, indicating that HMIP@A could effectively deplete GSH.

### 3.2. Enhanced Diffusion of HMIP@A

**Figure 3:**
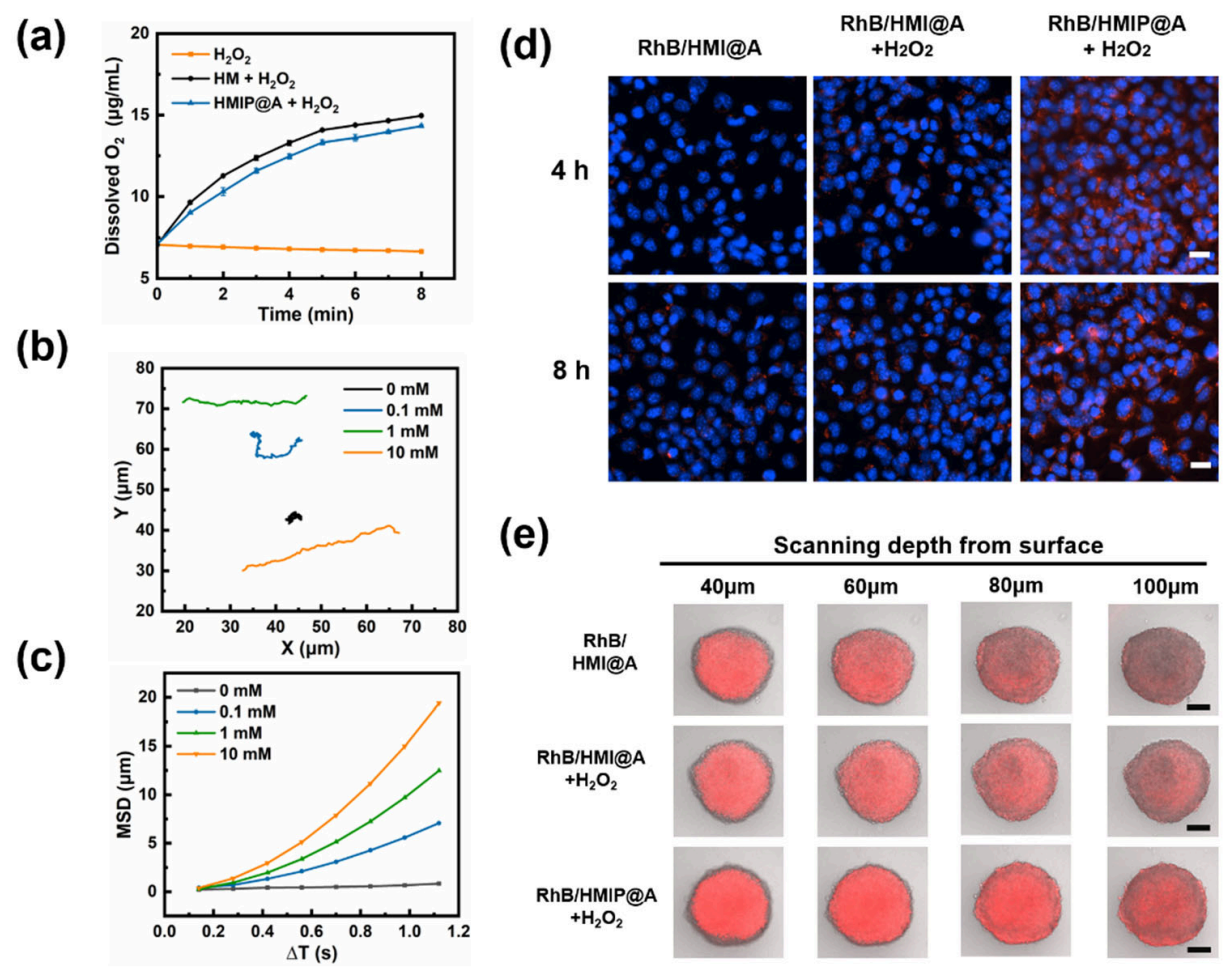
(**a**) O_2_ generation by HM and HMIP@A in 0.1 mM H_2_O_2_ within 8 min, *n* = 3. (**b**) Motion trajectories of HMIP@A at different H_2_O_2_ concentrations. (**c**) MSD plots of HMIP@A at different H_2_O_2_ concentrations. (**d**) Intracellular uptake of RhB/HMI@A, RhB/HMI@A+H_2_O_2_, and RhB/HMIP@A+H_2_O_2_, scale bar = 20 μm. (**e**) Z-stack image of tumor spheres from the top to the equator plane; scale bar = 100 μm.

First, a dissolved oxygen meter was used to evaluate the catalytic activities of HM and HMIP@A at equivalent HM concentrations (Figure 3a) [47]. The results demonstrated that in the presence of H_2_O_2_, both HM and HMIP@A generated comparable amounts of oxygen, indicating that the asymmetric PDA coating and aptamer conjugation did not impair catalytic activity, thereby supporting the motion capability of HMIP@A. Furthermore, the motion behavior of HMIP@A driven by H_2_O_2_ fuel was investigated. The motion trajectories of HMIP@A were recorded in real time using a microscope equipped with NIS-Elements AR 3.2 software. In the absence of H_2_O_2_, HMIP@A exhibited typical Brownian motion, characterized by random trajectories and low mean squared displacement (MSD) (Figure 3b,c) [48]. Upon the addition of H_2_O_2_, both the travel distance and the MSD of HMIP@A increased significantly in a concentration-dependent manner as the H_2_O_2_ concentration increased from 0.1 to 10 mM. These results confirm the H_2_O_2_-driven motion of HMIP@A, attributed to the localized oxygen gradient generated during the catalytic decomposition of H_2_O_2_.

To evaluate the effect of the nanomotor motion on cellular uptake, a Rhodamine B (RhB) fluorescence labeling strategy [22,49] was employed to prepare RhB/HMI@A and RhB/HMIP@A, which were subsequently incubated with H-4T1 cells. Fluorescence imaging showed that, upon the addition of H_2_O_2_, the fluorescence intensity of the RhB/HMI@A group showed a slight increase but remained nearly unchanged overall, whereas that of the RhB/HMIP@A group increased significantly (Figure 3d). These results demonstrate that nanomotor motion enhances cellular internalization.

To further evaluate penetration in multicellular spheroids, a multicellular tumor spheroid model was utilized. Spheroids composed of 4T1 cells and NIH/3T3 cells were constructed [20] to assess the penetration behavior of different nanoparticles (Figure 3e). The results showed that the RhB/HMIP@A+H_2_O_2_ group exhibited a significantly greater penetration depth than the RhB/HMI@A+H_2_O_2_ group. This enhanced penetration is attributed to the active self-propulsion of HMIP@A arising from its asymmetric structure, which facilitated deep transport within the dense multicellular architecture.

### 3.3. The Mechanism of the Interaction Between HMIP@A and Cells

**Figure 4:**
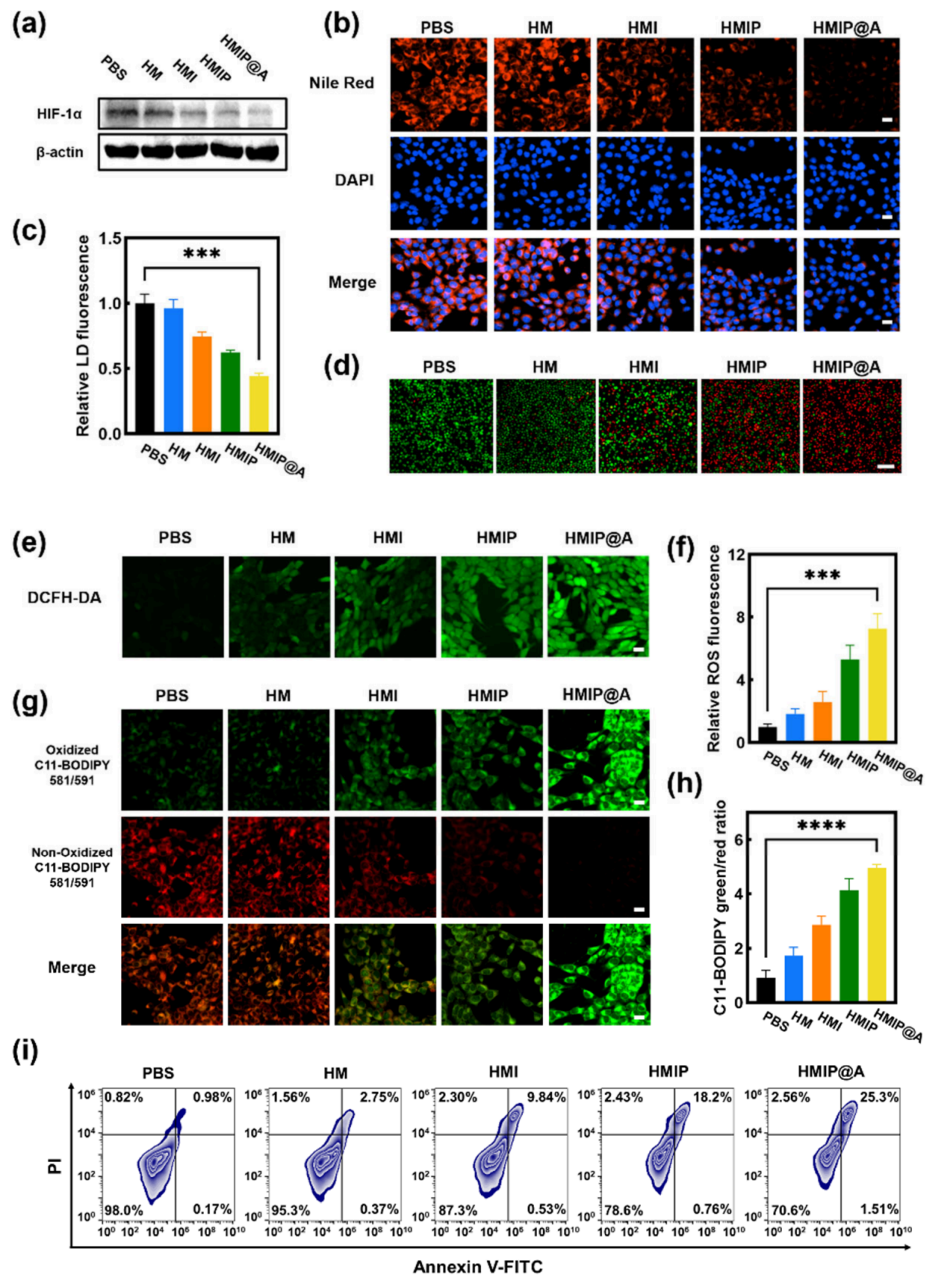
(**a**) Western blot analysis of HIF-1α expression. (**b**) Fluorescence imaging of LDs in H-4T1 cells after different treatments using Nile Red staining; scale bar: 20 μm. (**c**) Quantitative analysis of LDs corresponding to (**b**), *n* = 3. *** *p* < 0.001. (**d**) Fluorescence imaging of cells incubated after different treatments using Calcein-AM/PI staining; scale bar: 100 μm. (**e**) Fluorescence imaging of intracellular ROS in H-4T1 cells after different treatments using DCFH-DA staining; scale bar: 20 μm. (**f**) Quantitative analysis of ROS levels corresponding to (**e**), *n* = 3. *** *p* < 0.001. (**g**) Fluorescence imaging of LPO in H-4T1 cells after different treatments using C11-BODIPY 581/591 staining; scale bar: 20 μm. (**h**) Quantitative analysis of LPO corresponding to (**g**), *n* = 3. **** *p* < 0.0001. (**i**) Flow cytometry analysis of cell apoptosis after different treatments.

LDs are dynamic organelles for neutral lipid storage, and their intracellular accumulation is tightly regulated by HIF-1α. To investigate the effect of HMIP@A on LD formation in cells with elevated HIF-1α expression, the protein levels of HIF-1α were evaluated (Figure 4a). Compared with the PBS group, HIF-1α expression in the HMIP@A-treated group was significantly downregulated. This reduction is attributed to hypoxia alleviation via MnO_2_-catalyzed oxygen generation from endogenous H_2_O_2_, together with the inhibitory effect of the loaded HIF-1α inhibitor in H-4T1 cells [47]. Consistent with the decreased HIF-1α expression, LD formation was markedly reduced (Figure 4b,c). These results indicate that HMIP@A alleviated intracellular hypoxia and suppressed LD biogenesis.

Subsequently, to evaluate the nanomotor’s targeting ability, H-4T1 cells were incubated with RhB/HMIP or RhB/HMIP@A. After 6 h, cells treated with RhB/HMIP@A exhibited markedly higher red fluorescence intensity than those treated with RhB/HMIP (Figure S2a,b). These results indicate that the aptamer enhances targeted cellular uptake of the nanomotors.

Building upon this enhanced targeting capability, we systematically evaluated the in vitro cytotoxic effects of the nanomotors. First, to demonstrate the necessity of nanomotor-mediated delivery, an MTT assay was performed to assess the cytotoxicity of the free HIF-1α inhibitor over a concentration range of 0-50 μM. The results (Figure S3a) showed that the free inhibitor exhibited limited cytotoxicity, indicating that metabolic intervention alone is insufficient to induce robust cell death. In contrast, Calcein-AM/PI staining (Figure 4d) revealed that HMIP@A induced the most profound cell death. To further assess the generality of this strategy, MTT assays were conducted in MDA-MB-231 cells. The results (Figure S3b) demonstrated that HMIP@A exhibited significant cytotoxicity in MDA-MB-231 cells.

To elucidate the subcellular mechanisms underlying the observed cell death, intracellular oxidative stress was evaluated. Intracellular ROS were detected using the DCFH-DA probe [50,51,52,53]. The HMIP@A-treated group exhibited the strongest green fluorescence (Figure 4e,f), indicating the highest ROS generation. Excessive ROS disrupts intracellular redox homeostasis. Meanwhile, MnO_2_ reacts with intracellular GSH, leading to its direct consumption, and these combined effects result in the rapid depletion of GSH. As shown in Figure S4, intracellular GSH levels were significantly reduced following HMIP@A treatment. The depletion of GSH compromises the cellular antioxidant defense system. Consistently, Western blot (WB) analysis (Figure S5a) revealed a pronounced downregulation of glutathione peroxidase 4 (GPX4) in the HMIP@A-treated group. The inactivation of GPX4 renders cells highly susceptible to lipid peroxidation (LPO), a hallmark of ferroptosis [54,55,56]. Fluorescence imaging using the C11-BODIPY 581/591 probe (Figure 4g) showed that the HMIP@A group exhibited the strongest green fluorescence, corresponding to the highest level of LPO (Figure 4h). To further confirm the involvement of ferroptosis, a rescue experiment using the specific ferroptosis inhibitor Ferrostatin-1 (Fer-1) was performed [1,57]. Co-incubation with Fer-1 partially restored cell viability (Figure S5b), indicating that the HMIP@A-induced LPO contributes to ferroptosis-mediated cell death.

Furthermore, this profound oxidative stress not only damages the lipid membranes but also induces severe organelle dysfunction. A JC-1 staining assay was performed to assess the mitochondrial membrane potential (Figure S6a). Compared with the strong red fluorescence in the PBS group, HMIP@A-treated cells exhibited a marked shift from red to green fluorescence (Figure S6b), indicating mitochondrial depolarization. Ultimately, this cascade of subcellular events-including suppressed LD biogenesis, GPX4 inactivation, excessive LPO, and mitochondrial dysfunction—converges to induce cell death. As shown in Figure 4i, flow cytometry analysis revealed the highest apoptosis rate in the HMIP@A group, demonstrating its potent cytotoxic effect arising from the synergistic action of multiple mechanisms.

### 3.4. Immune Response of HMIP@A in the Cell

Under prolonged oxidative stress, the extent of mitochondrial DNA breaks can exceed the cellular repair capacity. The combined effect of the fragmented DNA and released Mn^2+^ activates the cGAS-STING pathway, thereby promoting immune signaling responses. Based on this, we systematically evaluated the DNA damage and the activation of downstream immune signaling pathways induced by the HMIP@A treatment.

**Figure 5:**
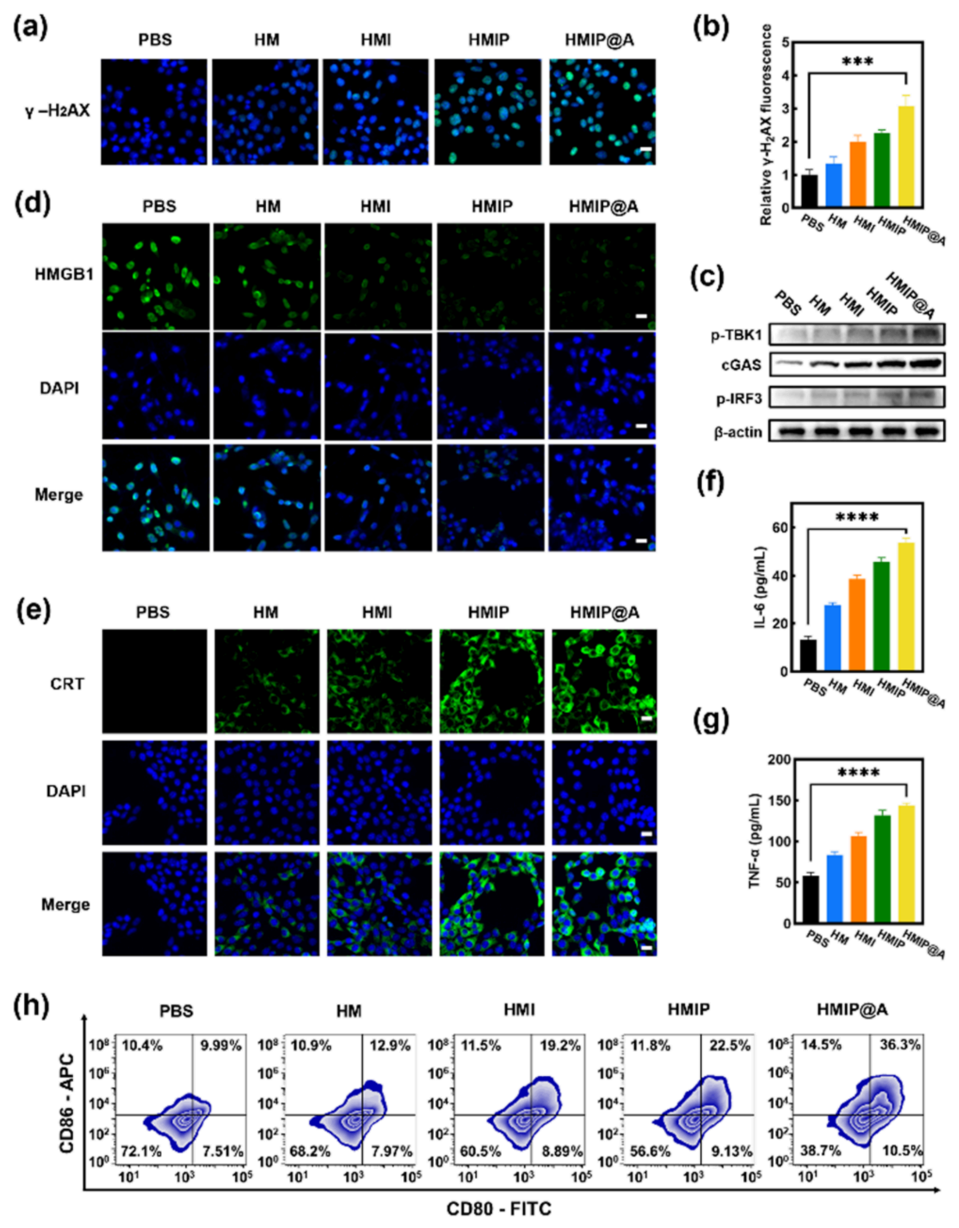
(**a**) Immunofluorescence images of γ-H_2_AX in H-4T1 cells after different treatments; scale bar: 20 μm. (**b**) Quantitative analysis of γ-H_2_AX fluorescence corresponding to (**a**), *n* = 3. *** *p* < 0.001. (**c**) Western blot analysis of the expression of p-TBK1, cGAS, and p-IRF3. Immunofluorescence images of (**d**) HMGB1 and (**e**) CRT in H-4T1 cells after different treatments; scale bar: 20 μm. The levels of cytokines of (**f**) IL-6 and (**g**) TNF-α released after different treatments, *n* = 3. **** *p* < 0.0001. (**h**) Flow cytometry analysis of DC2.4 cell maturation.

Immunofluorescence staining of γ-H_2_AX was performed to assess DNA damage. As shown in Figure 5a, the HMIP@A-treated group exhibited the highest γ-H_2_AX signal, indicating increased DNA damage (Figure 5b). Subsequently, the activation status of the cGAS-STING pathway was examined. WB analyses (Figure 5c) showed that cGAS expression was significantly upregulated following HMIP@A treatment. Concurrently, the levels of phosphorylated TBK1 (p-TBK1) and IRF3 (p-IRF3) [58] were also elevated, indicating activation of the cGAS-STING axis. To further validate the involvement of the cGAS-STING pathway, a rescue experiment was conducted using RU.521 [59,60,61], a specific inhibitor of cGAS. The secretion of interferon-β (IFN-β), a downstream effector of the cGAS-STING pathway, was quantitatively measured [12,62,63]. As shown in Figure S7, IFN-β levels were significantly increased in the HMIP@A-treated group, whereas this effect was markedly reduced upon co-treatment with RU.521. These results indicate that HMIP@A treatment activates the cGAS-STING pathway, thereby promoting IFN-β production.

The activation of the cGAS-STING pathway is typically accompanied by the release of damage-associated molecular patterns (DAMPs), a hallmark of ICD. We assessed the expression of high mobility group box 1 (HMGB1) and calreticulin (CRT) [64]. Following HMIP@A treatment, the intracellular level of HMGB1 was markedly decreased (Figure 5d). Concurrently, CRT fluorescence intensity was significantly increased, indicating CRT exposure on the cell surface (Figure 5e). This exposed CRT acts as an “eat me” signal, promoting the phagocytic uptake of dying cells by DCs.

After incubating H-4T1 cells with different nanoparticle treatments, the cell culture supernatants were collected and used to culture DC2.4 cells. Subsequently, ELISA assays [65] were performed to measure the secretion levels of inflammatory cytokines. The results showed that the levels of IL-6 and TNF-α were the highest in the HMIP@A-treated group, significantly higher than those in the other groups (Figure 5f,g). The maturation rate of DC2.4 cells was further determined by flow cytometry. The result showed that the maturation rate in the HMIP@A-treated group was higher than in the other groups (Figure 5h). These results indicate that the HMIP@A treatment enhances the immunogenicity of tumor cells, thereby promoting DC maturation [62].

In summary, the HMIP@A nanomotors synergistically induce oxidative cellular damage, trigger DNA damage, activate the cGAS-STING pathway, and promote the release of DAMPs. This cascade enhances cellular immunogenicity, demonstrating its potential as a synergistic immunotherapeutic platform.

## 4. Conclusions

In this study, we designed and constructed a multifunctional nanomotor, HMIP@A, to overcome the kinetic and metabolic limitations of conventional nanomedicines. The core innovation of this work lies in establishing a therapeutic strategy integrating “barrier disruption, enhanced oxidative damage, and immune activation.” First, to overcome the limited penetration associated with passive diffusion, the system employs an asymmetric PDA coating, partially exposing MnO_2_. Through the catalytic decomposition of endogenous H_2_O_2_ at these exposed sites, the nanomotor achieves active self-propulsion, enabling efficient transport within dense cellular architectures. Building upon this enhanced penetration, HMIP@A modulates the metabolic defense mechanisms of hypoxic tumor cells. Through a dual strategy combining MnO_2_-mediated oxygen generation and delivery of a small-molecule HIF-1α inhibitor, the nanomotors downregulate HIF-1α and suppress LD biogenesis. This modulation reduces the metabolic buffering capacity of tumor cells, limiting their ability to utilize LDs as antioxidant reservoirs to mitigate ROS-induced damage. Consequently, within this sensitized environment, ·OH generated via Mn^2+^-mediated Fenton-like reactions induces LPO and DNA damage. This cascade of events, driven by active motion and metabolic modulation, activates the cGAS-STING pathway, which, together with ICD, promotes DC maturation.

In summary, this study provides a multifunctional nanoplatform for drug delivery and therapy while demonstrating the potential of a strategy that integrates “modulating LD biogenesis to enhance oxidative sensitivity” with “activating the cGAS-STING pathway”. While the current work systematically elucidates the kinetic penetration behavior and subcellular mechanisms in vitro, we acknowledge that the lack of in vivo validation is a limitation of this study. The complex hemodynamic barriers, systemic clearance, and dynamic immune interactions in solid tumors represent factors that cannot be fully recapitulated by in vitro models. Therefore, future studies will focus on evaluating this nanomotor strategy in animal models, with particular emphasis on pharmacokinetics, long-term biosafety, and systemic antitumor immune responses. Ultimately, this work provides insights into overcoming microenvironmental barriers and supports the development of clinically translatable nanotherapeutic strategies.

## Data Availability

The original contributions presented in this study are included in the article/Supplementary Materials. Further inquiries can be directed to the corresponding authors.

## Supplementary Materials

The following supporting information can be downloaded at: https://www.mdpi.com/article/10.3390/bios16050261/s1. Figure S1: Stability of HMIP@A in PBS (pH 7.4); Figure S2: (a) Intracellular uptake of RhB/HMIP and RhB/HMIP@A; scale bar = 20 μm. (b) Fluorescent intensity corresponding to the orange line; Figure S3: (a) Cell viability of H-4T1 cells after treatment with HIF-1α inhibitor at different concentrations. (b) Cell viability of MDA-MB-231 cells after different treatments; Figure S4: Relative intracellular GSH levels in H-4T1 cells after different treatments, n = 3. **** *p* < 0.0001; Figure S5: (a) Representative Western blot analysis of GPX4. (b) Cell viability of H-4T1 cells after different treatments; Figure S6: (a) Fluorescence images of H-4T1 cells after different treatments using JC-1 staining; scale bar: 10 μm. (b) Quantitative analysis of fluorescence intensity corresponding to (a), *n* = 3. **** *p* < 0.0001; Figure S7: The levels of cytokines of IFN-β released after different treatments, *n* = 3. ** *p* < 0.01; Figure S8: Uncropped image of Figure 4a; Figure S9: Uncropped image of Figure 5c; Figure S10: Uncropped image of Figure S5a.
