# Supplementary material for: Imaging Study of MnO2-Based Nanomotors Modulating HIF-1α/Lipid Droplet Biogenesis and Activating the cGAS-STING Pathway
Source: Biosensors (Basel). 2026 May 1;16(5):261. doi: 10.3390/bios16050261 (PMC13204648; doi:10.3390/bios16050261)
Supplement: Supplementary file 1 [file biosensors-16-00261-s001.zip › biosensors-4221965-supplementary.pdf]

biosensors

MDPI

Supplementary Information

Imaging Study of MnO_2-Based Nanomotors Modulating HIF-1α/Lipid Droplet Biogenesis and Activating the cGAS-STING Pathway

Ziyi Li *, Yingxin Tian, Gefei Ren and Yingshu Guo *

School of Chemistry and Chemical Engineering, Qilu University of Technology (Shandong Academy of Sciences), Jinan, 250353, China
* Correspondence: 10431231018@stu.qlu.edu.cn (Z. L.); yingshug@126.com (Y. G.)

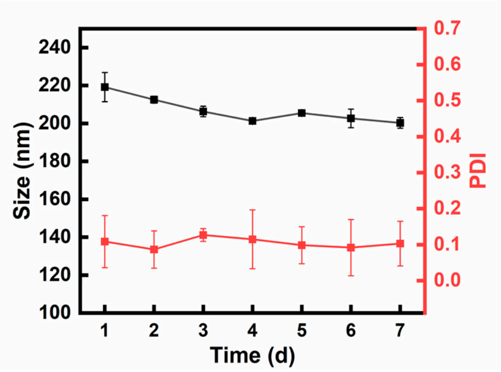

Figure S1. Stability of HMIP@A in PBS (pH 7.4).

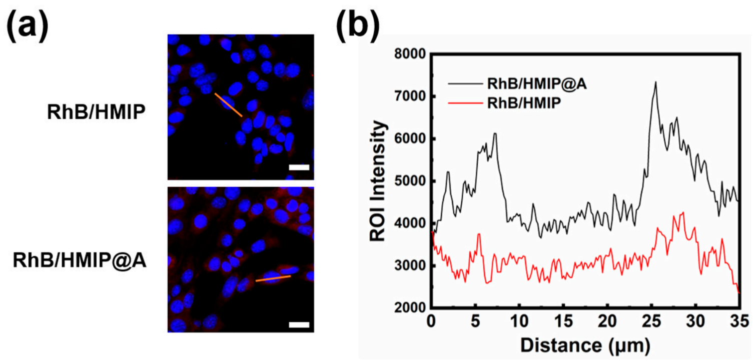

Figure S2. (a) Intracellular uptake of RhB/HMIP and RhB/HMIP@A, scale bar = 20 μm. **(b)** Fluorescent intensity corresponding to the orange line.

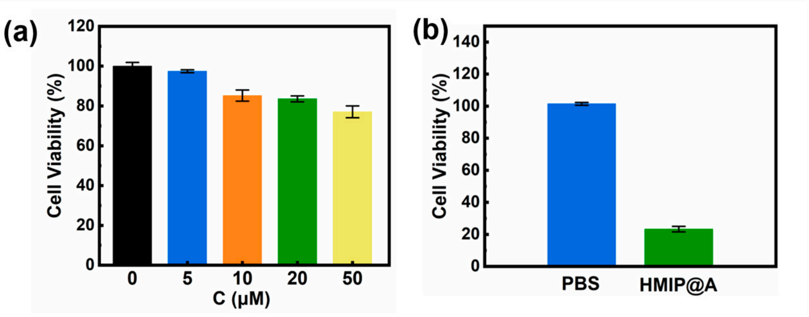

Figure S3. (a) Cell viability of H-4T1 cells after treatment with HIF-1α inhibitor at different concentrations. **(b)** Cell viability of MDA-MB-231 cells after different treatments.

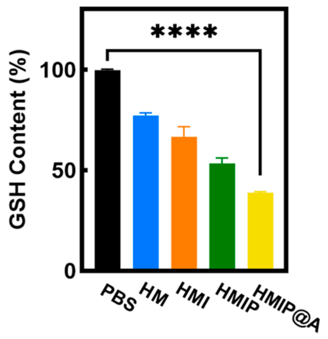

Figure S4. Relative intracellular GSH levels in H-4T1 cells after different treatments, $n = 3$. ****p < 0.0001.

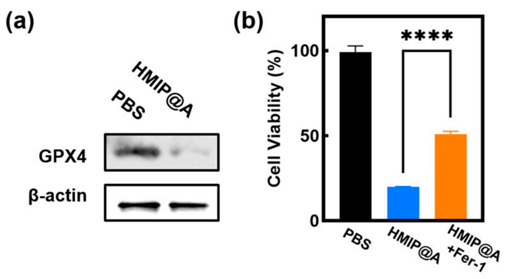

Figure S5. (a) Representative Western blot analysis of GPX4. **(b)** Cell viability of H-4T1 cells after different treatments.

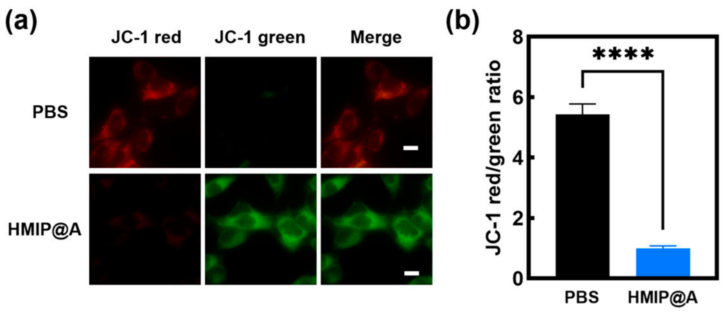

Figure S6. (a) Fluorescence images of H-4T1 cells after different treatments using JC-1 staining, scale bar: 10 μm. **(b)** Quantitative analysis of fluorescence intensity corresponding to (a), $n = 3$. ****p < 0.0001.

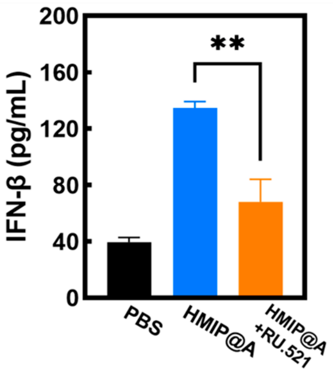

Figure S7. The levels of cytokines of IFN-β released after different treatments, $n = 3$. **p < 0.01.

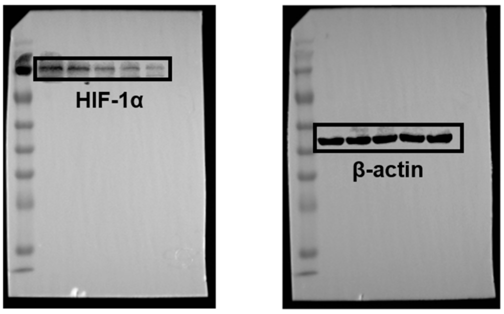

Figure S8. Uncropped image of Figure 4a.

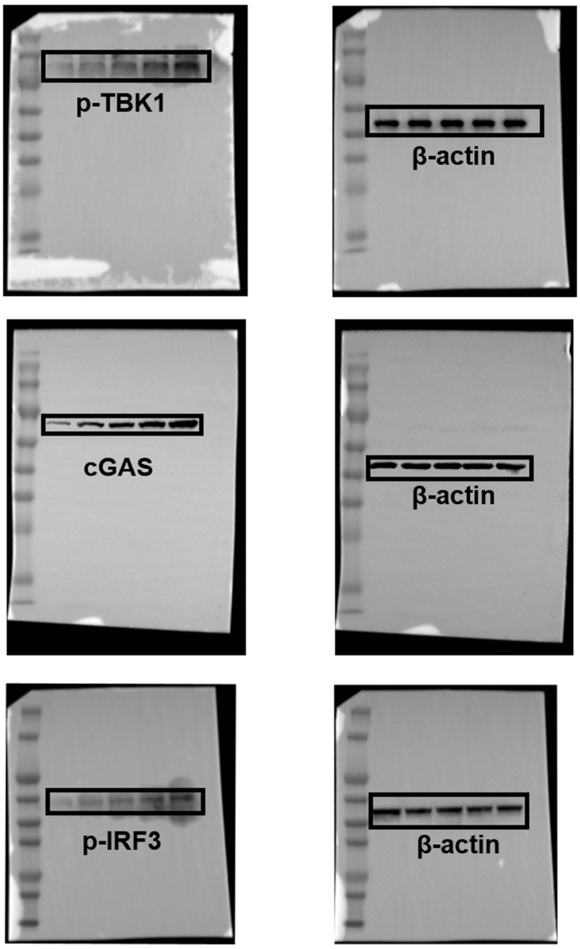

Figure S9. Uncropped image of Figure 5c.

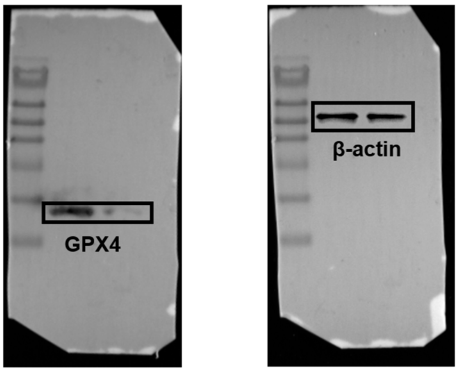

Figure S10. Uncropped image of Figure S5a.
